# Palatal Rugoscopy: A Tool for Ethnicity and Gender Identification Among Saudi and Kuwaiti Populations

**DOI:** 10.7759/cureus.52333

**Published:** 2024-01-15

**Authors:** Nishath Sayed Abdul, Jumana Abdullah Alzahrani, Sarah Saad Alharbei, Aldanah Tawfiq Almutib, Reem Abdullah Ibnjuma, Zainab Hammad Almutairi

**Affiliations:** 1 Faculty of Oral Pathology, Department of Oral and Maxillofacial Surgery & Diagnostic Sciences, College of Medicine and Dentistry, Riyadh Elm University, Riyadh, SAU; 2 General Dentistry, College of Medicine and Dentistry, Riyadh Elm University, Riyadh, SAU

**Keywords:** forensic dentistry, gender identification, kuwaiti population, saudi population, palatal rugae

## Abstract

Background

The establishment of human identity has always been a concern after mass disasters, and the role of odontology in forensics has greatly evolved. Nowadays, palatal rugoscopy is a widely used method in the recognition of human identity due to its uniqueness in the course, direction, length, form, position, and enduring nature against disintegration. Its easy applicability, cost-effectiveness, and prompt results can be applied to festering, scorched bodies and in the absence of missing upper limbs and fingers. This study was undertaken to evaluate palatal rugoscopy as a tool to recognize human identity and gender between two different ethnic populations.

Methodology

A cross-sectional comparative study was conducted in the Department of Oral Maxillofacial Surgery and Diagnostic Sciences, Riyadh Elm University, Riyadh, Saudi Arabia, to recognize ethnicity and gender among the Saudi and Kuwaiti populations. A total of 364 participants were selected from the outpatient department between September 2022 and December 2022. All 364 participants were distributed into two groups after the application of inclusion and exclusion criteria. After obtaining informed consent from all study participants, study models were prepared for final interpretation. The outlining of rugae was done with the help of a sharp graphite pencil, and the assessment of various parameters, including total number, length, direction, unification, and shape of rugae, was done. A comparison was made between the two populations. SPSS version 26.0 (IBM Corp., Armonk, NY, USA) was employed to assess variations in the mean values of both the total number and the distribution of rugae on the right and left sides across different ethnic groups and genders.

Results

In this study, a total of 364 participants were included. Of the total participants, 184 were Saudis and 180 were Kuwaitis, with 188 males and 176 females. A comparative evaluation of rugae among ethnic groups showed that Saudi participants had a mean number of 8.92 ± 0.660 palatal rugae, whereas in Kuwaiti participants it was 8.68 ± 0.649 (p = 0.001). When rugae length was assessed between genders among Saudi participants, it was found that the majority of participants had primary rugae with a length of more than 5 mm, and the difference was statistically significant (p = 0.002). The majority of Saudi males had forwardly positioned rugae, while the majority of Saudi females had more backwardly placed rugae. When rugae length was assessed in males and females among Kuwaiti participants, it was found that males had more primary rugae than females. This study found that the majority of Kuwaiti males had a wavy shape of rugae, while the majority of Kuwaiti females had more straight rugae.

Conclusions

This study concluded that among the two ethnic groups, the total number, length, direction, unification, and shape of rugae were different between genders, with significant differences in some parameters. Therefore, palatal rugoscopy might be useful as a tool to recognize gender and ethnicity and may provide better results when used as an additional tool along with other dependable forensic tools.

## Introduction

The establishment of human identity is always a concern in forensics, especially after a disaster, because identification of a dead person necessitates a cautious assessment of DNA, fingerprinting, dental records, and all other forms of postmortem verifications [1,2]. Although DNA testing is a powerful tool for establishing human identity, it is very expensive and requires intense time and labor. In addition to this, recovering DNA from human remains after a disaster or mishap is difficult and has no framework without any direct reference to the sample or family sample for further evaluation. Therefore, methods of human identification have evolved into methods such as fingerprinting and odontological comparisons [3]. Forensic dentistry entails medicolegal recognition of a person through dental findings [4]. Forensic dentistry chiefly uses human dentition due to its resistance to external and internal factors. Visual identification, fingerprinting, DNA matching, and human dentition are frequently used methods of identification in forensics, but when data regarding these are unavailable or unclear, palatal rugoscopy is used [1,2].

Palatal rugs are defined as irregular, uneven fibrous tissue folds located on the anterior one-third of the roof of the mouth, either one or equal, on the other side of the mid-palatine rapha. The investigation and study of palatal rugae is called *Rugoscopy*, which was primarily coined by Thomas Hermosa in 1932 [5]. Palatal rugae are the unique identity of every individual. This uniqueness is present in the course, direction, length, form, and position. The rugae can withstand disintegration up to seven days after death, and the shape of the rugae does not alter with age. Rugaes are protected by teeth, lips, cheeks, tongue, and a buccal pad of fat against trauma and fire and are less affected by surgery, chemicals, and heat. If destroyed, they are reproducible at the same site [6]. Palatal rugae have special attention in the case of edentulous arches, and rugoscopy has expanded because of its application in cases where no fingerprints are obtainable [2]. Nowadays, palatal rugoscopy is a widely used method in the recognition of human identity due to its applicability to festering, scorched bodies missing upper limbs and fingers. It also has the advantage of being a speedy, easy, and reasonably priced method [7].

Thomas and Kotze performed a detailed analysis of rugae patterns among different South African populations and concluded that rugae patterns were inimitable among different ethnic groups and could be productively used as a standard for genetic investigations [8]. After that, various studies were performed to understand the morphological pattern of rugae and its association with ethnicity and gender [9-11]. However, very little data is available for the comparison between Saudi and Kuwaiti populations. Both Saudi Arabia and Kuwait are Arab nations, and a significant proportion of their populations identify as Arab. However, despite these similarities, there are some distinctions in terms of race or ethnicity between the two countries. Both Saudi Arabia and Kuwait have diverse tribal structures that contribute to variations in regional identities and affiliations. Tribes may have specific traditions, dialects, and cultural practices, leading to differences in the ethnic makeup within each country. While Arabs constitute the majority in both countries, there are also non-Arab communities in each nation. For example, in Saudi Arabia, there are sizable expatriate communities from various countries, including South Asian, Southeast Asian, and African populations. In Kuwait, expatriates comprise a significant proportion of the population, contributing to a diverse ethnic landscape. Therefore, this study was conducted to assess palatal rugoscopy as a tool for ethnicity and gender identification among Saudi and Kuwaiti populations.

## Materials and methods

A descriptive comparative study was conducted in the Department of Oral Maxillofacial Surgery and Diagnostic Sciences, Riyadh Elm University, Riyadh, Saudi Arabia to recognize ethnicity and gender among the Saudi and Kuwaiti populations. Ethical clearance was obtained from the Institutional Ethical Committee of Riyadh Elm University (approval number: FRP/2022/456/845/795).

Sample size estimation

For this study, a sample size of 364 was determined, ensuring a statistical power of 80% and a significance level of 5%. This robust sample size was selected to ensure sufficient statistical precision and reliability for detecting potential differences in palatal rugae patterns between ethnicity and gender.

Eligibility criteria

Individuals between 18 and 25 years old who willingly consented to participate in the study were included. This age range included individuals who had completed their growth and development but were within an age range where the palatal rugae patterns were likely to remain relatively stable. Participants provided self-identified information regarding their ethnicity. Individuals from migrant populations were excluded from the study. Individuals with any kind of prosthesis, removable or fixed partial dentures, braces, lip, and palatal anomalies, including cleft left lip and palate, were excluded from the study.

Data collection

Study models were prepared after taking an alginate impression of a maxillary arch for final interpretation. The outlining of the rugae was done using a sharp graphite pencil. The assessment of various parameters, including the distribution of the total number of rugae (right and left sides), length of rugae, direction of rugae, total number of unifications of rugae, and shape of rugae, was assessed according to the Thomas and Kotz classification [8]. Palatal impressions were inspected by two examiners. Before the examination, both examiners underwent a calibration process to ensure consistency and reliability in their assessments. This calibration involved training sessions and discussions to standardize their approach to palatal rugoscopy. Interrater reliability was 0.88, indicating good agreement between the two examiners. The value of 0.88 suggests a high level of consistency in their observations, reinforcing the reliability of the palatal rugoscopy assessments performed by the examiners.

In this study, the length of rugae was divided into the following two categories: (i) primary rugae: length greater than 5 m; and (ii) secondary rugae: length between 3 and 5 m. Individual palatal rugae on the dental cast were located and identified. Reference points, including the origin (starting point), midpoint, and termination (ending point) of each ruga, were established. The linear distance between the selected points on each ruga was determined using a digital caliper. Three categories of rugae were considered for the assessment of rugae direction, namely, forward, backward, and perpendicular. The orientation of each ruga was established by assessing the angle formed between the line connecting their origin and insertion and a line perpendicular to the mid-palatine raphe. Rugae were categorized as forward-directed (F) if the angle was less than 90° (acute), backward-directed (B) if the angle exceeded 90° (obtuse), and perpendicular (P) if the angle was precisely 90°, aligning with the midpoint between the origin and insertion.

Unification occurs when the fusion of two rugae occurs at the site of origin or termination. Unification is assessed under the categories of converging rugae and diverging rugae. A diverging pattern is characterized by two rugae that originate from the same point but then spread apart horizontally. Conversely, a converging pattern is observed when two rugae originate from distinct regions and then come together horizontally. The contour or shape of rugae was assessed under the following four categories: straight, wavy, curved, and circular. Rugae that were in a straight line from the start point until insertion were considered straight. Rugae that had gentle hemispherical shapes were considered curved. Rugae that had a serpentine contour were considered wavy, whereas rugae with a definite continuous ring configuration were considered circular [8].

Statistical analysis

Data were transferred to spreadsheets and analyzed using SPSS software version 26.0 (IBM Corp., Armonk, NY, USA). The mean value of the total number of rugae and the distribution of rugae on the right and left sides were compared using the Student’s t-test, whereas the association of length, direction, unification, and rugae shape was assessed using the chi-square test. A p-value less than 0.05 was considered significant.

## Results

A total of 364 participants were included in the study. Among them, 184 were Saudis, and 180 were Kuwaitis, with 188 males and 176 females. No significant difference was noted between genders for the Saudi and Kuwaiti ethnicities (p = 0.670), as seen in Table 1, suggesting matched groups.

**Table 1:** Number of study participants. NS = not significant

Gender	Saudi, n (%)	Kuwaiti, n (%)	Total	Chi-square statistic	P-value
Male	93 (25.5%)	95 (26.1%)	188 (51.6%)	0.182	0.670 (NS)
Female	91 (25%)	85 (23.4%)	176 (48.4%)		
Total	184 (50.5%)	180 (49.5%)	364 (100%)		

A comparative evaluation of rugae among ethnic groups showed that the mean value of the total number of rugae among Saudi participants was 8.92 ± 0.660. In Kuwaiti participants, it was 8.68 ± 0.649, with a significant difference (p = 0.001). However, no significant difference was found in the right and left sides of rugae distribution among both ethnic groups, as shown in Table 2.

**Table 2:** Comparative evaluation of rugae between the groups. * = significant; NS = not significant

Sides	Saudi population		Kuwaiti population		t statistic	P-value
	Mean	SD	Mean	SD		
Right	4.43	0.497	4.39	0.489	0.888	0.375 (NS)
Left	4.27	0.776	4.18	0.861	-1.017	0.310 (NS)
Total	8.92	0.660	8.68	0.649	3.508	0.001*

A comparative evaluation of rugae among genders between ethnic groups showed that the mean value of the total number of rugae in Saudi males was 8.86 ± 0.604, whereas, in Kuwaiti males, it was 8.73 ± 0.626, with no significant difference. The mean value of the total number of rugae in Saudi females was 8.97 ± 0.706 and in Kuwaiti females was 8.62 ± 0.672, with a statistically significant p-value of 0.001, as seen in Table 3.

**Table 3:** Genderwise comparative evaluation of rugae between ethnic groups. * = significant; NS = not significant

Sides	Saudi male		Kuwaiti male		t statistic	P-value
	Mean	SD	Mean	SD		
Right	4.42	0.497	4.35	0.479	1.073	0.285 (NS)
Left	4.01	0.883	4.22	0.746	-1.760	0.080 (NS)
Total	8.86	0.604	8.73	0.626	1.471	0.143 (NS)
Sides	Saudi female		Kuwaiti female		t statistic	P-value
	Mean	SD	Mean	SD		
Right	4.45	0.500	4.44	0.499	0.202	0.840 (NS)
Left	4.37	0.798	4.33	0.808	0.715	0.715 (NS)
Total	8.97	0.706	8.62	0.672	3.300	0.001*

When rugae length was assessed in males and females among Saudi participants, the majority of participants had primary rugae with a length of more than 5 mm, and the difference was statistically significant (p = 0.002). The majority of Saudi males had forwardly positioned rugae, while Saudi females had more backwardly placed rugae (p = 0.001). When unification was assessed, more converging rugae were found in Saudi males, while Saudi females had more diverging rugae (p = 0.001). This study found that the majority of Saudi participants had more curved shapes of rugae, but the difference between males and females was not significant, as shown in Table 4.

**Table 4:** Rugae length, direction, unification, and shape patterns in both genders among Saudi participants. * = significant; NS = not significant

Saudi participants				
	Males, n (%)	Females, n (%)	Chi-square statistic	P-value
Rugae length				
Primary	90 (96.7%)	76 (83.5%)	9.160	0.002*
Secondary	3(3.2%)	15 (16.4%)		
Direction of rugae				
Forward	48 (51.6%)	27 (29.6%)	14.171	0.001*
Backward	36 (38.7%)	38 (41.7%)		
Perpendicular	09 (9.6%)2	26 (28.5%)		
Unification of rugae				
Converging	57 (61.2%)	32 (35.1%)	12.571	0.001*
Diverging	36 (38.7%)	59 (64.8%)		
Shape of rugae				
Straight	04 (4.3%)	06 (6.5%)	0.541	0.910 (NS)
Wavy	35 (37.6%)	35 (38.4%)		
Curved	45 (48.3%)	42 (46.1%)		
Circular	09 (9.6%)	08 (8.7%)		

When rugae length was assessed in males and females among Kuwaiti participants, it was found that males had more primary rugae than females, but the difference was not significant. Similarly, no significant difference was found in the position of rugae among males and females in Kuwait. When unification was assessed, more converging rugae were found in Kuwaiti males, while Kuwaiti females had more diverging rugae (p = 0.004). The study found that the majority of Kuwaiti males had the wavy shape of rugae, while the majority of Kuwaiti females had more straight rugae, which was significant (p = 0.04), as shown in Table 5.

**Table 5:** Rugae length, direction, unification, and shape patterns in both genders among Kuwaiti participants. * = significant; NS = not significant

Kuwaiti participants				
	Males, n (%)	Females, n (%)	Chi-square statistic	P-value
Rugae length				
Primary	71 (74.7%)	57 (67%)	1.287	0.257 (NS)
Secondary	24 (25.2%)	28 (33%)		
Direction of rugae				
Forward	26 (27.7%)	23 (27%)	0.301	0.860 (NS)
Backward	54 (56.8%)	46 (54.1%)		
Perpendicular	15 (15.7%)	16 (18.8%)		
Unification of rugae				
Converging	66 (69.4%)	41	8.394	0.004*
Diverging	29 (30.5%)	44		
Shape of rugae				
Straight	18 (18.9%)	32 (37.6%)	8.318	0.04*
Wavy	32 (33.6%)	20 (23.5%)		
Curved	23 (24.2%)	19 (22.3%)		
Circular	22 (23.1%)	14 (16.4%)		

**Figure 1:**
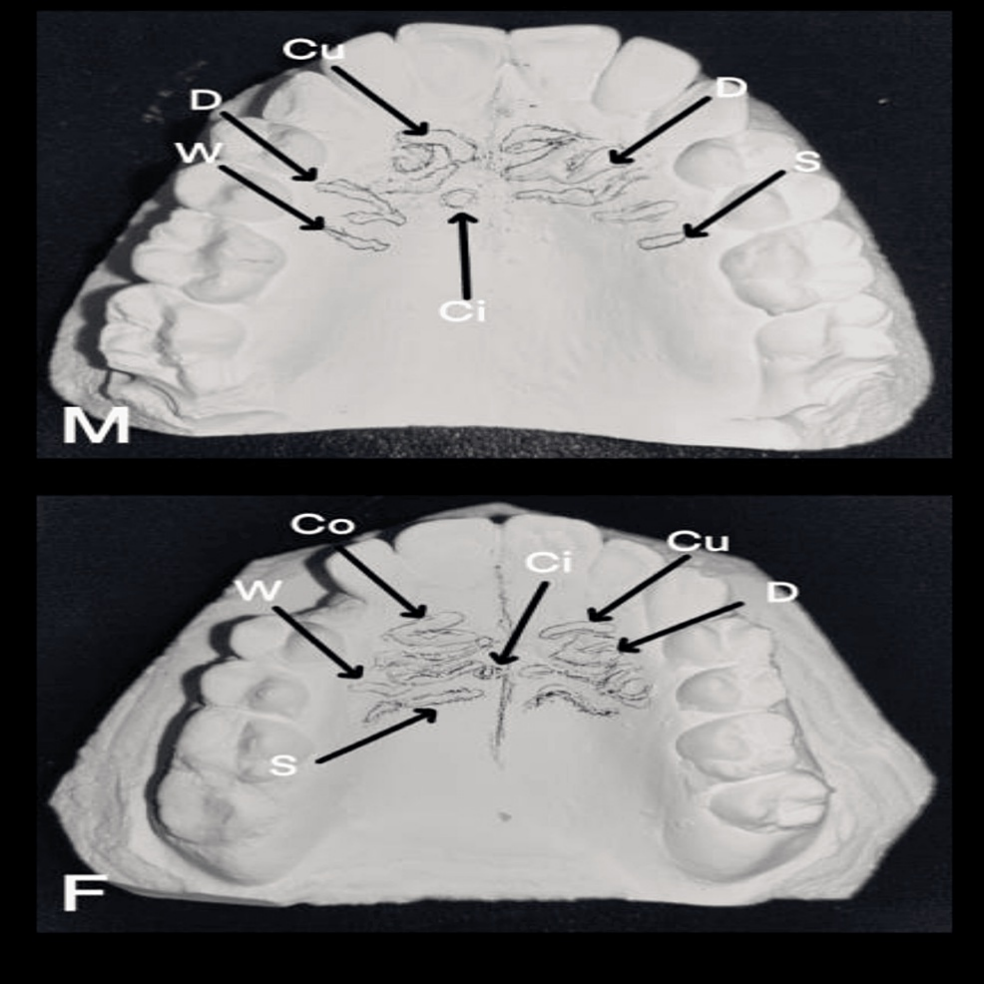
Saudi male and female participants. Different types of palatal rugae shapes delineated in two maxillary dental casts of Saudi participants (male and female) S = straight; W = wavy; Cu = curved; Ci = circular; Co = converging unification; D = diverging unification

**Figure 2:**
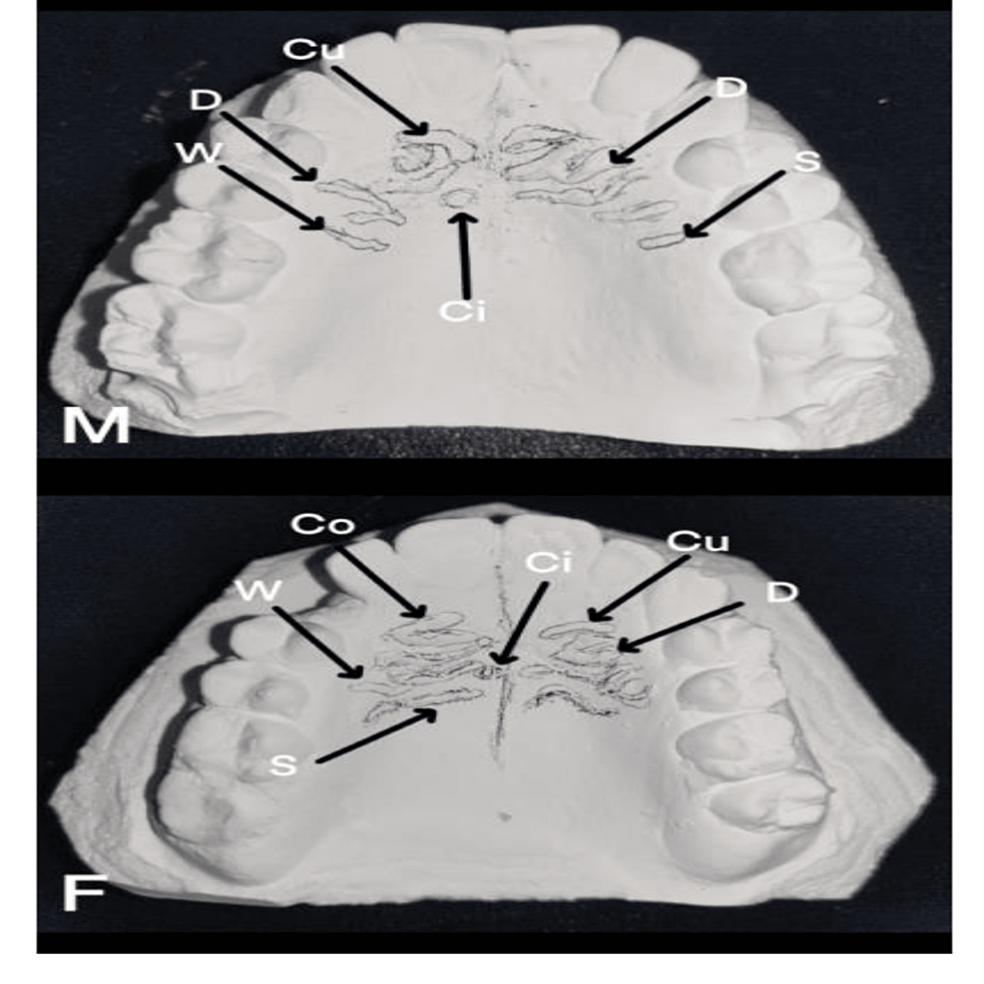
Kuwaiti male and female participants. Different types of palatal rugae delineated in the maxillary casts of Kuwaiti participants (male and female). S = straight; W = wavy; Cu = curved; Ci = circular; Co = converging unification; D = diverging unification

When rugae length was assessed between the two ethnic groups, Saudi participants had more primary rugae than Kuwaiti participants, and this difference was statistically significant (p = 0.001). In this study, Saudi participants had more forwardly placed rugae, while Kuwaiti participants had more backwardly positioned rugae (p = 0.008). More diverging rugae were found in the Saudi population, whereas more converging rugae were found in the Kuwaiti population (p = 0.034). When the shape of rugae was assessed among both groups, the majority of Saudi participants had curved rugae, whereas the majority of Kuwaiti participants had wavy rugae, with a significant p-value of 0.001, as shown in Table 6. Figure 1 shows the dental casts of Saudi participants depicting the various rugae patterns. Figure 2 shows the dental casts of Kuwaiti participants showing various rugae presentations.

**Table 6:** Rugae length, direction, unification, and shape patterns among Saudi and Kuwaiti participants. * = significant

	Saudi participants, n (%)	Kuwaiti participants, n (%)	Chi-square statistic	P-value
Rugae length				
Primary	166 (90.2%)	128 (71.1%)	21.384	0.001*
Secondary	18 (9.7%)	52 (28.8%)		
Direction of rugae				
Forward	75 (40.7%)	49 (27.2%)	9.536	0.008*
Backward	74 (40.2%)	100 (55.5%)		
Perpendicular	35 (19%)	31 (17.2%)		
Unification of rugae				
Converging	89 (48.3%)	107 (59.4%)	4.491	0.034*
Diverging	95 (51.6%)	73 (40.5%)		
Shape of rugae				
Straight	10 (5.4%)	50 (27.7%)	51.794	0.001*
Wavy	70 (38%)	52 (28.8%)		
Curved	87 (47.2%)	42 (23.3%)		
Circular	17 (9.2%)	36 (20%)		

## Discussion

The application of odontology in forensics has evolved. Due to various distinctive characteristics, rugae can be used for recognizing human identity in the absence of other tools. This study was conducted to establish palatal rugoscopy as a tool for ethnicity and gender among Saudi and Kuwaiti populations in the Department of Oral Maxillofacial Surgery and Diagnostic Sciences, Riyadh Elm University, Riyadh, Saudi Arabia. In this study, a total of 364 individuals participated, and various parameters of rugae were assessed among both ethnic groups and genders.

Population

In this study, significant differences were noted in the mean number of rugae among the Saudi and Kuwaiti populations. Similarly, Hosmani et al. reported that total rugae were higher in Indian populations compared to Tibetan populations [12]. In contrast, according to Shubha et al., no differentiation exists in the total number of rugae among the north and south Indian populations [13]. In this study, Saudi participants had more forwardly placed rugae, while Kuwaiti participants had more backwardly positioned rugae. When the shapes of rugae were compared, Saudi participants had more curved rugae, whereas Kuwaiti participants had more wavy rugae. A study by Hosmani et al. found the leading patterns of rugae among Indians were straight and wavy, whereas it was curved in Tibetans [12]. When the shape of rugae was assessed among two Indian populations, the predominant shape among the north Indian cohorts was curved, and in the south Indian population, it was wavy or circular [13].

When the unification of rugae was assessed, more diverging rugae were found in the Saudi population, whereas more converging rugae were found in the Kuwaiti population, with a significant difference. Similar results were found in a study by Hauser et al., wherein Swazi and Greek populations had significant differences in rugae patterns. The authors concluded that the growth of rugae depends upon the growth of the palate [14]. In this study, the rugae patterns of Saudi and Kuwaiti populations were different in terms of total number, length, unification, and shape and, therefore, can be used for the recognition of human identity. English et al. also concluded that the pattern of palatal rugae can be successfully used as a characteristic pattern to discriminate between individuals [1]. However, according to Alshammari et al., the length of palatal rugae can be useful in recognizing the age group, but it has limited exactness [15].

Gender

When rugae patterns were assessed and a comparison was made between males and females of Saudi and Kuwaiti populations, the mean value of the total number of rugae was higher in Saudi males compared with Saudi females and Kuwaiti males compared with Kuwaiti females. Similar results were noted in a study by Fawzi et al. During the comparison, males in Saudi Arabia had more rugae than females [16]. In contrast, Kumawat et al. found that the total number and prototype of rugae were not linked with gender, while only the length of rugae was associated with gender [17].

Saudi and Kuwaiti males had more primary rugae than females. Similar results were reported in the study by Babaji et al., who found that a higher number of primary rugae were present among boys than girls, and the difference was notable [18]. Oberoi et al. also found that the mean number of palatal primary rugae was considerably higher among males (7.52 ± 2.67) compared to females (6.43 ± 1.91) [19]. In the study by Swetha et al., the length of rugae (primary rugae) was greater in males, and females had more secondary and tertiary rugae [20]. Contradictory findings were reported by Pillai et al., where there was no difference in palatal rugae between males and females [21].

Saudi males had a higher number of forwardly directed and converging rugae, whereas Saudi females had a higher count of backwardly directed and diverging rugae. Similarly, Pramanik et al. found that females had more backwardly positioned rugae and males had more perpendicular rugae, but no disparity was found in the figure of the unifications of rugae among both genders [22]. Similar results were found by Pereira et al., who found that males had more converging rugae and females had more diverging rugae [23]. According to Alshammari et al., the backward course and circular shape of rugae were noticeable in both genders among the Saudi population, and females had more backward-directed rugae compared with males. In this study, Kuwaiti males had more backwardly placed rugae; similarly, Dwivedi et al. found that the backward course of rugae was common in males [24]. In this study, Saudi males and females had more curved rugae, whereas Kuwaiti males had more wavy rugae, and Kuwaiti females had more straight rugae. Whereas in a study by Alshammari et al., the circular shape of rugae was more common among males. They concluded that the direction and shape of rugae are closely linked with gender [16].

Limitations

This study lacks longitudinal data, which could provide insights into how palatal rugae patterns evolve over time. Longitudinal studies are essential for understanding the stability of these patterns and their potential changes with age or environmental factors.

## Conclusions

The comparative analysis of palatal rugae characteristics between Saudi and Kuwaiti participants revealed statistically significant differences. Saudi participants exhibited a higher prevalence of primary rugae, forwardly placed rugae, and diverging rugae, while Kuwaiti participants demonstrated more backwardly positioned rugae, converging rugae, and wavy rugae. Additionally, the shape of rugae differed significantly between the two groups, with curved rugae being predominant among Saudi participants and wavy rugae among Kuwaiti participants. Therefore, palatal rugoscopy might be useful as a tool to recognize gender and ethnicity, and it may give better results when used as an additional means along with other dependable forensic tools. In this study, a comparison was made between the two populations sharing similar geographical conditions. Moreover, as the sample size of this study was small, continued research with diverse and larger population sizes in comparison to different ethnic groups is recommended for more precise results. These findings suggest distinctive palatal rugae patterns that may be ethnically associated, highlighting the potential utility of palatal rugoscopy in ethnicity identification among Saudi and Kuwaiti populations.
